# Macroscopic Fluorescence Imaging: A Novel Technique to Monitor Retention and Distribution of Injected Microspheres in an Experimental Model of Ischemic Heart Failure

**DOI:** 10.1371/journal.pone.0101775

**Published:** 2014-08-04

**Authors:** Andreas Martens, Sebastian V. Rojas, Hassina Baraki, Christian Rathert, Natalie Schecker, Sara Rojas Hernandez, Kristin Schwanke, Robert Zweigerdt, Ulrich Martin, Shunsuke Saito, Axel Haverich, Ingo Kutschka

**Affiliations:** 1 Department of Cardiothoracic, Transplantation and Vascular Surgery, Hannover Medical School, Hannover, Germany; 2 Leibniz Research Laboratories for Biotechnology and Artificial Organs, Hannover Medical School, Hannover, Germany; 3 Department of Anaesthesiology and Intensive Care Medicine, Hannover Medical School, Hannover, Germany; Sapienza University of Rome, Italy

## Abstract

**Background:**

The limited effectiveness of cardiac cell therapy has generated concern regarding its clinical relevance. Experimental studies show that cell retention and engraftment are low after injection into ischemic myocardium, which may restrict therapy effectiveness significantly. Surgical aspects and mechanical loss are suspected to be the main culprits behind this phenomenon. As current techniques of monitoring intramyocardial injections are complex and time-consuming, the aim of the study was to develop a fast and simple model to study cardiac retention and distribution following intramyocardial injections. For this purpose, our main hypothesis was that macroscopic fluorescence imaging could adequately serve as a detection method for intramyocardial injections.

**Methods and Results:**

A total of 20 mice underwent ligation of the left anterior descending artery (LAD) for myocardial infarction. Fluorescent microspheres with cellular dimensions were used as cell surrogates. Particles (5×10^5^) were injected into the infarcted area of explanted resting hearts (*Ex vivo myocardial injetions* EVMI, n = 10) and *in vivo* into beating hearts (*In vivo myocardial injections* IVMI, n = 10). Microsphere quantification was performed by fluorescence imaging of explanted organs. Measurements were repeated after a reduction to homogenate dilutions. Cardiac microsphere retention was 2.78×10^5^±0.31×10^5^ in the EVMI group. In the IVMI group, cardiac retention of microspheres was significantly lower (0.74×10^5^±0.18×10^5^; *p<0.05*). Direct fluorescence imaging revealed venous drainage through the coronary sinus, resulting in a microsphere accumulation in the left (0.90×10^5^±0.20×10^5^) and the right (1.07×10^5^±0.17×10^5^) lung. Processing to homogenates involved further particle loss (*p<0.05*) in both groups.

**Conclusions:**

We developed a fast and simple direct fluorescence imaging method for biodistribution analysis which enabled the quantification of fluorescent microspheres after intramyocardial delivery using macroscopic fluorescence imaging. This new technique showed massive early particle loss and venous drainage into the right atrium leading to substantial accumulation of graft particles in both lungs.

## Introduction

Recently, an increasing number of studies have been conducted, aiming at regenerating infarcted myocardium by injecting cells into the damaged area [1]–[7]. Direct intramyocardial injection is an appealing technique, as the infarcted area can be targeted with high local cell concentrations in comparison to intracoronary application [8], [9]. Cardiac stem cell transplantation has been investigated in numerous randomized clinical studies [10]–[13]. However, low graft size following cell injection is commonly observed and has been critically discussed in several studies [14]–[20]. Mechanisms of cell loss are low cell retention [15], [21], variable biodistribution [19], [22], [23] and poor graft survival [24]. However, recent studies suggest that major cell loss occurs directly after injection and is explained by application aspects [16], [19], [21], [25]. Therefore, strategies that improve the retention, biodistribution and survival of injected cells are being developed [26]. For this purpose, imaging techniques that evaluate cell fate *in vivo* are essential. However, quantifying cell retention in myocardial tissue involves time consuming processing and generally require expensive imaging methods such as positron emission tomography (PET), magnetic resonance imaging (MRI) or computerized tomography (CT) [16]–[18], [27], [28]. Additionally, millions of trackable cells need to be produced for every experiment, which adds further costs and complexity to the experimental setting. In order to address this challenge, fluorescent microspheres have been successfully used as cell surrogates to monitor cardiac retention following intramyocadial injections with similar loss patterns [15], [25]. Microspheres are broadly available and can be tracked by several methods including FACS analysis and macroscopic fluorescence imaging. Teng et al. reported early microsphere loss of more than 90% after beating heart injections (in vivo) in Lewis rats [25]. In their study, microspheres were detected using fluorescence flow cytometry, which involved chemical and mechanical reduction of treated organs to homogenate dilutions. However, this procedure still has some limitations. Firstly, organ distribution cannot be analyzed by this method. Secondly, reduction to homogenates might involve further particle loss. Thirdly, the method remains time consuming in practice and cannot be applied to monitor injection success in ongoing experiments. Therefore, our aim was to develop a reliable analytical method to monitor intramyocardial injections. The main hypothesis of the present study was that direct macroscopic fluorescence imaging would provide reliable measurements for particle detection and quantification following intramyocardial injections. Measurements were performed on intact organs following intramyocardial injection and after reduction to homogenate dilutions. Applying our novel technique, we compared cardiac microsphere retention and distribution patterns following beating heart injections (*in vivo*) and non-beating heart injections (*ex vivo*).

## Materials and Methods

### Animal Care

Balb/c mice (Charles River Laboratories, Germany) (n = 20) were subdivided into two groups: *ex vivo* microsphere injection (EVMI) into non-beating harvested hearts (n = 10) and *in vivo* intramyocardial microsphere injection (IVMI) into beating hearts (n = 10). Each animal underwent myocardial infarction by ligature of the left anterior descending coronary artery (LAD) as described previously [2]. This study was carried out in strict accordance with the recommendations from the Guide for the Care and Use of Laboratory Animals of the German Society for Medical Research (ETS123). The study was also approved by the State Animal Studies Committee (Permit Number: 10-0079). All surgery was performed under anesthesia and all efforts were made to minimize suffering.

### Microsphere preparation

Non-biodegradable, fluorescent polystyrene microspheres of 10-µm diameter (Fluorospheres, Beckman Coulter, USA; light emission of 525–700 nm wavelengths when excited at 488 nm) were used as cell surrogates. Particle size was chosen considering regular human cell sizes. Aliquots of 5.0×10^5^ particles were prepared in 15 µl of Phosphate buffered saline (PBS) for each injection.

### Microsphere quantification

Microsphere concentration in the stock solution (1 ml samples, n = 10) was analysed using an automated particle counting device (CASY, Roche Innovatis, Germany). Dilutions ranging from 2.50×10^5^ to 0.16×10^5^ microspheres in 4 ml PBS were generated on 6-well plates (n = 8 for each concentration) and standard curves for macroscopic fluorescence imaging were established using an IVIS Lumina II device (Xenogen - Caliper LifeSciences, USA). Macroscopic fluorescence imaging of organ homogenates (4 ml in PBS) was performed in 6-well plates after thorough resuspension. Direct macroscopic fluorescence imaging of non-homogenized organs was performed after placing the organs in a standard non-fluorescent culture dish.

### Injection technique

In the *EVMI* group, hearts were perfused with cardioplegia solution ten minutes after LAD ligation and were harvested. Microsphere injection was perfomed immediately *ex vivo*. In the IVMI group, animals underwent beating heart microsphere injection 10 minutes after initiating myocardial infarction *in vivo*. Hearts and lungs were harvested ten minutes after injection. For intramyocardial injection, the needle was inserted subepicardially into the left ventricle (LV) lateral to the LAD and distal to the ligature. A single bolus was injected into the myocardium using a 50-µl Microliter Syringe (Hamilton, USA) equipped with a 33-gauge needle. Injection volumes remained consistent at 15-µl. The puncture site was compressed with a small surgical sponge during withdrawal of the needle. A successful injection was defined by immediate tissue blanching in the surrounding area of the injection site.

### Generating tissue homogenates

To quantify the organ dose after microsphere injection, organs were homogenized as described previously. Organs were washed in PBS and Radioimmuno Precipitation Assay (RIPA) buffer [50 mM Tris-HCl, ph 7,4, 1% NP-40, 0,25% Na-deoxycholate, 150 mM NaCl] was added before the organs were homogenized with an Ultra-Turrax (IKA, Germany) device for 1 minute. All instruments were systematically rinsed with RIPA buffer and the follow-through was collected in order to minimize potential microsphere loss. Subsequently, samples were stored for 24 hours at room temperature to complete tissue lyses. Filtration was performed (40 µm filter, BD Falcon, USA) to remove tissue debris; additional buffer (10 ml) was washed through the filter to avoid further microsphere loss. To recover microspheres, follow-through was recovered via centrifugation (4000 rcf for 5 min) and the pellets containing microspheres were diluted in 4 ml PBS and transferred into 6-well plates for macroscopic fluorescence quantification.

### Statistical analysis

The linear correlation coefficient [Pearson, (r)] was determined to compare fluorescence signal and microsphere count in standard dilutions. All results are presented as mean ± standard deviation (SD). Comparison of group values between two groups was performed with Student's t–test. For the comparison of multiple groups, oneway ANOVA was used, followed by Fisher's least significant difference (LSD) test or Dunnett's t-test, as appropriate. Differences were considered significant at p<0.05. Statistical analyses were performed using GraphPad Prism version 5 for Mac OS X, GraphPad Software, USA.

## Results

### Validation of microsphere quantification

**Figure 1:**
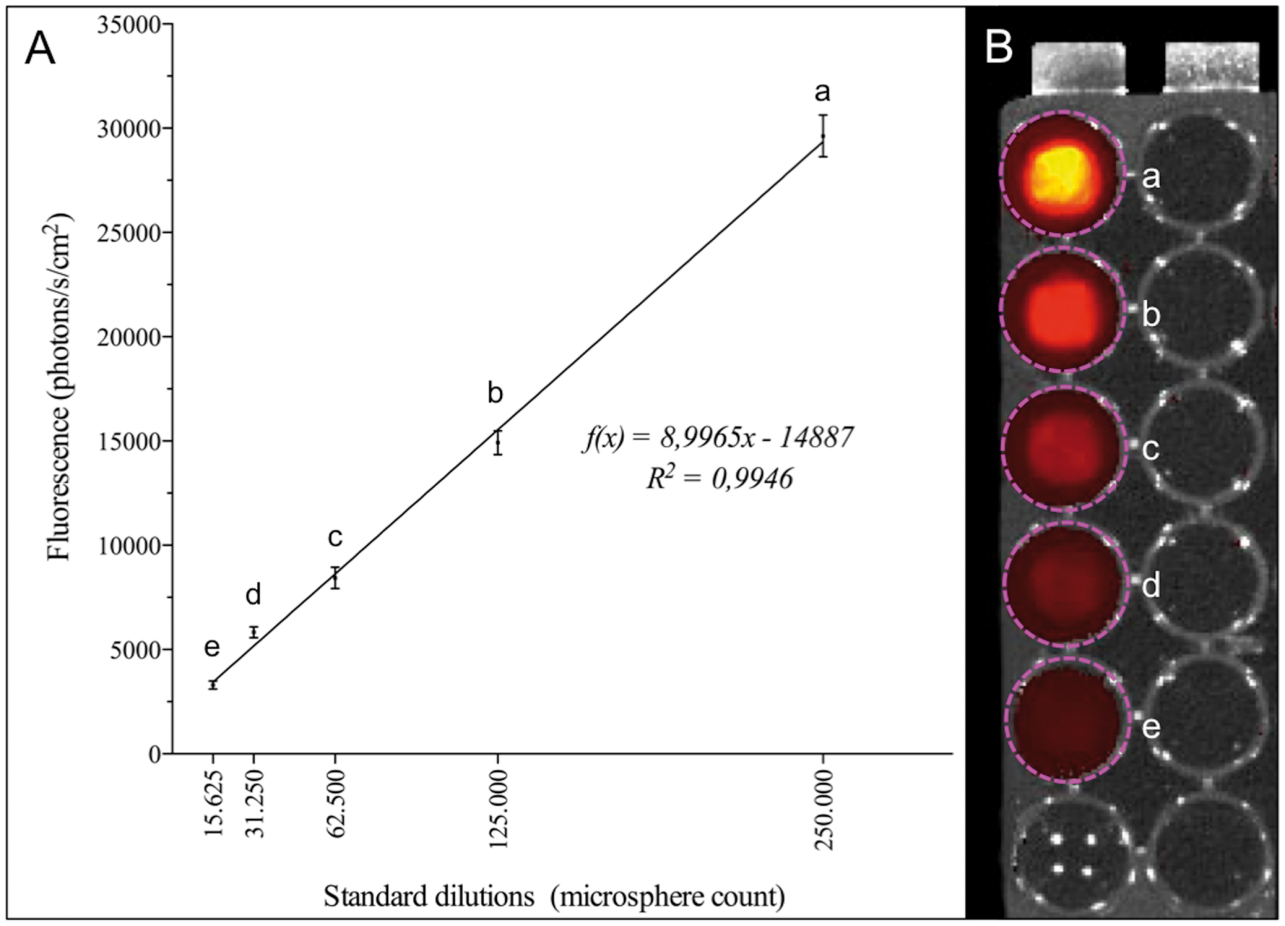
Fluorescence analysis of standard microsphere dilutions. **A** Correlation between microsphere amounts and fluorescence signals, which allows the calculation of microsphere concentrations in the function of fluorescence signals. **B** Standard dilutions between 250.000 (a) and 15.625 (e) were arranged on 96-well plates.

Automated particle count of standardized dilutions with a nominal amount of 1×10^6^ revealed a mean of 0,99×10^6^±7.0×10^4^ microspheres. IVIS quantification was performed using reference dilutions: The correlation coefficient (r) between absolute microsphere counts (CASY) and fluorescence intensity was r = 0,997 (*p<0.001*) for fluorescence analysis. The mean fluorescence values of microsphere standard dilutions are shown in Table 1 and Fig. 1.

**Table 1:** Overview of fluorescence intensitites of standard microsphere dilutions.

microspheres	X	15625	31250	62500	125000	250000
**Fluorescence**	**mean**	3297	5832	8432	14921	29630
	**SD**	189	260	511	576	996
	**n**	8	8	8	8	8

### *Ex vivo* microsphere injection

**Figure 2:**
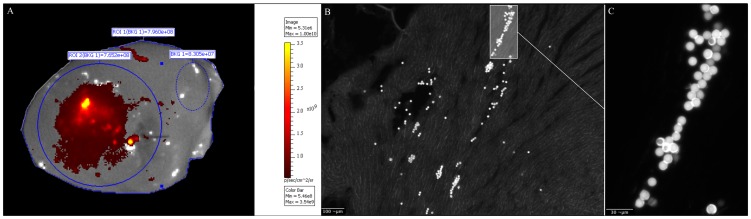
Ex vivo microsphere injection (EVMI). **A** Macroscopic fluorescence imaging of a murine heart following injection of 5×10^5^ microspheres. The injection zone (blue circle) shows low (dark red) to high (yellow) microsphere concentrations. For proper fluorescence analysis, background fluorescence (dotted blue circle) must be assessed on every image. **B** Histological assessment of a murine heart after microsphere injection **C** Augmentation of the microsphere distribution passage inside the left ventricle (LV).

**Figure 3:**
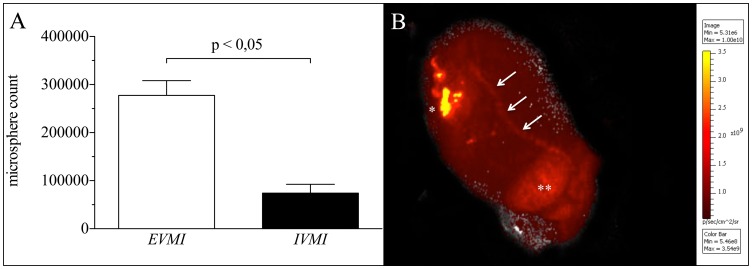
Myocardial microsphere concentrations and venous drainage of microspheres. **A** Comparision of myocardial microsphere concentrations after injection *ex vivo* (EVMI) and *in vivo* (IVMI). **B** Myocardial fluorescence analysis unveils venous drainage (arrows) from the injection zone (*) to the right atrium (**).

Macroscopic fluorescence analysis of the *EVMI group* showed a calculated mean equivalent to 2,78×10^5^±3,06×10^4^ microspheres that remained inside the target myocardium (55,5±6,1% of injected particles) (Fig. 2). The imaging analysis showed a venous drainage from the injection site through the coronary sinus into the right atrium (Fig. 3). Homogenate dilutions of these hearts contained 1,52×10^5^±1,57×10^4^ (30,4±3,1%) microspheres. Analysis of the filters used during the homogenization process revealed 1,06×10^5^±9,06×10^3^ (21,3±1,8%) microspheres. Overall, a 44,5% loss of microspheres was measured after non-beating ex vivo injection.

### *In vivo* microsphere injection

**Figure 4:**
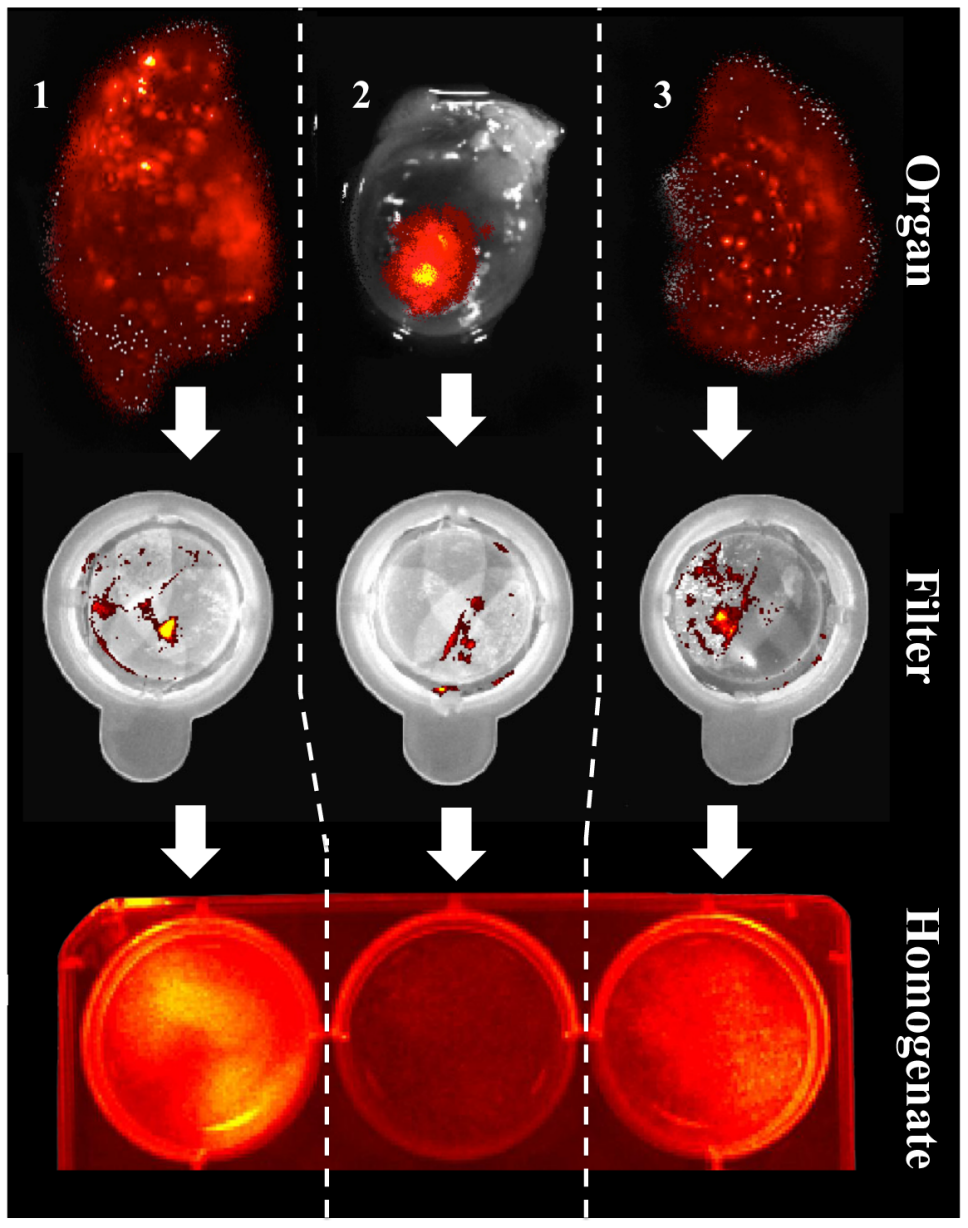
Distribution of microspheres after injection in vivo (IVMI). The IVMI-group showed distribution to both lungs (right lobe: 1 – left lobe: 3) 10 minutes after injection into the heart (2). Additionally, the homogenization (Organ – Filter – Homogenate) process involved filter retention of microspheres leading to lower microsphere counts in homogenate dilutions.

**Figure 5:**
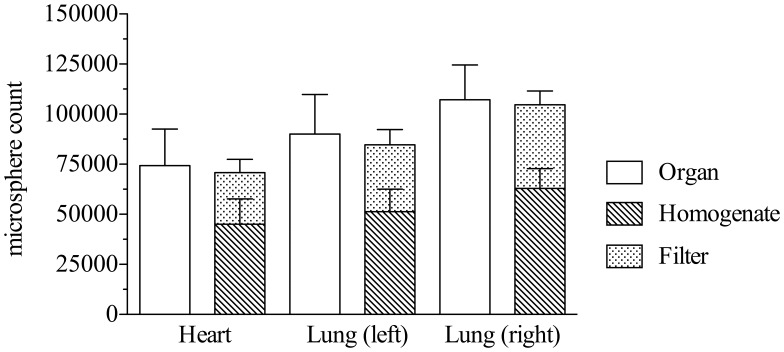
Overview of microsphere quantification in the IVMI group. Of half a million microspheres which were up taken by the syringe, only few (<75.000) remain in the injection site after 10 minutes of beating heart action. Additionally, further homogenization will lead to lower particle amounts. This phenomenon should be considered when quantifying microspheres in homogenate dilutions. The overall summation of fluorescence signals, retained in filters and organ homogenates respectively, resulted in comparable microsphere counts to whole organ IVIS measurements before homogenization.

After *in vivo* injection 7,43×10^4^±1,83×10^4^ (14,9±3,7%) microspheres were retained in the myocardium, in the left lung 9,00×10^4^±1,97×10^4^ (18,0±5,9%) and in the right lung 10,72×10^5^±1,74×10^4^ (21,4±3,5%) (Fig. 4). Compared to these quantification results, microsphere content in homogenate dilutions was lower resulting in 4,51×10^4^±1,27×10^4^ (9,0±2,5%) microspheres in the heart, 5,12×10^4^±1,13×10^4^ (10,3±2,3%) in the left lung and 6,30×10^4^±9,81×10^4^ (12,6±2,0) in the right lung. Again, filtering caused significant loss of 2,56×10^4^±6,65×10^4^ microspheres (5,2±1,3%; *p<0.05*) of heart tissue, 3,34×10^4^±7,63×10^4^ (6,7±1,5,%; *p<0.05*) of left lung tissue and 4,17×10^4^±6,87×10^4^ (8,3±1,4%; *p<0.05*) of right lung tissue. Notably, the overall summation of fluorescence signals, retained in filters and organ homogenates respectively, resulted in comparable microsphere counts to whole organ IVIS measurements before homogenization (Fig. 5).

## Discussion

Cell therapy has been discussed as a promising approach to regenerate damaged myocardium [29]. Currently, different cell types are being critically examined in small animal models with regard to their regenerative potential, safety and survival in host organisms [30]. The mouse model is a well-established first step in clinical translation. In mice, the most frequently applied technique of intramyocardial cell delivery is a direct injection in infarcted myocardium. However, inadequate implantation efficiency of donor cells remains a major drawback and affects the systematic evaluation of cellular transplant effects [19]. A key observation is the rather low cell graft size in histological assessments compared to the preoperatively measured number of injected cells. Early after transplantation, they were estimated to be only in a one-digit percent range of the transplanted cells [14]. Therefore, methods capable of monitoring intramyocardial injections are needed to investigate injection losses. An overview of different imaging methods for monitoring intramyocardial injections is shown in Table 2. Teng et al. showed that the largest loss occurs directly after injection by means of mechanical washout and injection failure [25]. In order to abstract from cell survival, these authors applied fluorescent microspheres as cell surrogates. After intramyocardial injection in rats and piglets, organs were harvested and reduced to homogenate dilutions for microsphere quantification by FACS analysis. Teng et al. found less than 10% of the expected microspheres in the explanted hearts directly after injection, supporting the mechanical washout hypothesis. Nevertheless, there may be other explanations for these results, prompting us to ask the following questions:

**Table 2:** Advantages and disadvantages of different imaging methods for monitoring of intramyocardial injections.

Method	Exemplary target	Advantages	Disadvantages
**SPECT**	Cells labelled with radionuclides [16], [18]	High sensitivity, available for human use, available for large animals, assessment of graft viability, 3D mapping (anatomy)	Potentially hazardous, poor image resolution, high costs, poor availability
**MRI**	Cells labelled with iron oxides [27], [28]	High resolution, avoids ionizing radiation, combination with other (functional) measurements, 3D mapping (anatomy), small and large animals	Potential signal decay in longitudinal studies, high costs, poor availability, no assessment of graft viability
**BLI**	Cells labelled with Luciferase [4], [5], [6], [19]	High sensitivity, longitudinal studies, assessment of graft viability	Poor resolution, only 2D mapping (anatomy), no clinical translation, only small animals, gene transfer needed
**MFI**	Fluorescent microspheres [15], [25]	Cell-free, inexpensive, fast and easy to perform, good availability, in vivo and ex vivo assessment, quantification feasible	Potential autofluorescence, only small animals, limited to preliminary studies

BLI: Bioluminescence Imaging; MFI: Macroscopic Fluorescence Imaging; MRI: Magnetic Resonance Imaging; SPECT: Single Photon Emission Computed Tomography.

1. Which mechanisms – instead or in addition to post-injection wash out – are mainly responsible for low microsphere retention in the heart?
2. Can fluorescent microspheres reliably be quantified by macroscopic fluorescence imaging in whole organs? Are there systematic errors related to microsphere quantification in organ homogenates?
3. Are microspheres distributed to other organs, in particular into the lungs, shortly after injection?

The first challenge of this study was to develop a fast and reliable method for microsphere quantification using macroscopic fluorescence imaging. Following the experimental design, 5×10^5^ microspheres were injected into explanted hearts. This quantity reflects the cell amounts that are commonly applied in cell-therapy studies [1]. The obtained results show that the quantity of microspheres found in organ explants was much lower than expected. The imaging technique enabled us to illustrate how particles were drained from the injection site to the right atrium, a mechanism that also explains pulmonary accumulation of injected microspheres when injections are performed in beating hearts. This amount of loss was similar to what has been shown in cellular experiments [18]. In order to ensure that macroscopic fluorescence imaging was capable of detecting the total amount of microspheres contained in each organ, quantifications were also performed after a tissue reduction process. Microsphere quantification in homogenate dilutions revealed significantly lower particle amounts compared to the analysis of whole organs. However, a fluorescence analysis of filters applied for tissue homogenisation unveiled significant microsphere leftovers in the filter. This finding suggests that assessment of graft retention in homogenates is prone to a systematic error of the method, resulting in the underestimation of microsphere numbers. On the other hand, the sum of detected particles in homogenate dilutions and filters equalled the number of quantified microspheres in intact organs. Thus, the main hypothesis was confirmed: Macroscopic fluorescence imaging can be used to reliably analyze microsphere concentrations in intact organs, overcoming the drawbacks related to organ homogenisation.

Considering the massive microsphere loss *ex vivo*, further loss *in vivo* was expected. The quantification of microspheres confirmed this hypothesis and revealed significantly lower means in particle concentrations in murine hearts. This finding can be explained by the beating heart, which increases venous drainage leading to microsphere accumulation in both lungs [16].

### Limitations

This study has some limitations. Firstly, the behavior of the fluorescent microspheres after injection into the myocardium might be not be identical to that of the cells. The microspheres, unlike the cells, do not have surface adhesion molecules or plasticity in shape, which may play a role in retention rate. Nevertheless, the rationale for using microspheres that correspond closely in size to the implanted cells is to remove the possibility of cell death from the biological factors, thus isolating the mechanical and technical mechanisms of cell loss. Secondly, there is also a limitation concerning the use of a small animal model. Because of the small size of the heart, the relative thinness of the ventricular wall, and rapid cardiac contractions, mechanical loss may be more pronounced in smaller animals than in larger animals or in clinical settings. The mouse model was chosen in this case because it has been used extensively in basic research on myocardial cell therapy, and is an important gatekeeper in clinical translation.

## Conclusions

In this study, we developed a fast and simple technique capable of monitoring the retention and biodistribution of cell surrogates in a common experimental model of myocardial infarction. Macroscopic fluorescence imaging provided reliable and reproducible particle quantifications of injected microspheres following intramyocardial injections. Thus, this technique enabled us to assess treated organs giving an optical feedback of microsphere distribution. The imaging method can also be applied to observe and quantify these effects in cell grafts when cells are marked properly (i.e. Luciferase detection using bioluminescence imaging) [19]. Thus, the method is suited for studies that aim at improving the retention and biodistrubition of injected cells by initially investigating new strategies on the accessible microsphere model before performing complex cell experiments. For myocardial injections, it has also been demonstrated by others that massive particle loss occurs immediately after administration [16], [19]. This fact underlines the need for monitoring and standardization of surgical injection procedures using novel techniques.
